# Erratum: A case report of two Kala-azar cases in China diagnosed by metagenomic next-generation sequencing

**DOI:** 10.3389/fmed.2022.1067340

**Published:** 2022-10-27

**Authors:** Frontiers Production Office

**Affiliations:** Frontiers Media SA, Lausanne, Switzerland

**Keywords:** kala-azar, *Leishmania*, MNGs, fever, diagnosis

The authors and publisher would like to note that this article was originally published in *Frontiers in Medicine*. As of 21 October 2022 this manuscript has now been transferred to *Frontiers in Microbiology* with the doi: 10.3389/fmicb.2022.922894.

